# Accurate Identification of DNA Replication Origin by Fusing Epigenomics and Chromatin Interaction Information

**DOI:** 10.34133/2022/9780293

**Published:** 2022-10-29

**Authors:** Fu-Ying Dao, Hao Lv, Melissa J. Fullwood, Hao Lin

**Affiliations:** ^1^Center for Informational Biology, University of Electronic Science and Technology of China, Chengdu 610054, China; ^2^School of Biological Sciences, Nanyang Technological University, Singapore 639798, Singapore; ^3^Cancer Science Institute of Singapore, National University of Singapore, 14 Medical Dr, Singapore 117599, Singapore; ^4^Department of Molecular Life Sciences, University of Zurich, Winterthurerstrasse 190, 8057 Zurich, Switzerland; ^5^Institute of Molecular and Cell Biology, Agency for Science, Technology and Research (A∗STAR), Singapore 138673, Singapore

## Abstract

DNA replication initiation is a complex process involving various genetic and epigenomic signatures. The correct identification of replication origins (ORIs) could provide important clues for the study of a variety of diseases caused by replication. Here, we design a computational approach named iORI-Epi to recognize ORIs by incorporating epigenome-based features, sequence-based features, and 3D genome-based features. The iORI-Epi displays excellent robustness and generalization ability on both training datasets and independent datasets of K562 cell line. Further experiments confirm that iORI-Epi is highly scalable in other cell lines (MCF7 and HCT116). We also analyze and clarify the regulatory role of epigenomic marks, DNA motifs, and chromatin interaction in DNA replication initiation of eukaryotic genomes. Finally, we discuss gene enrichment pathways from the perspective of ORIs in different replication timing states and heuristically dissect the effect of promoters on replication initiation. Our computational methodology is worth extending to ORI identification in other eukaryotic species.

## 1. Introduction

**Figure 1:**
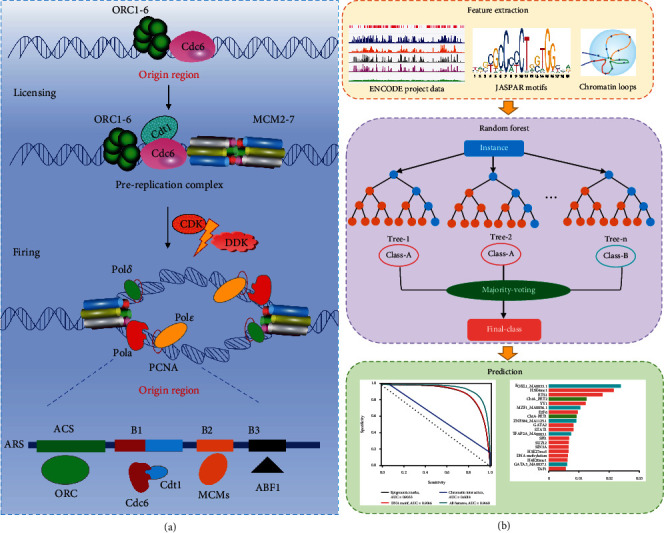
Formation and activation of DNA replication origins and the workflow of this study. (a) Origin licensing: during G1 phase of the cell cycle, the origin recognition complex (ORC) binds DNA and, together with Cdt1 and Cdc6, loads minichromosome maintenance complexes (MCM), the core motor of the replicative helicase, as inactive head-to-head double hexamers (MCM-DHs) around double-stranded DNA. Origin firing: during S phase, CDK2 and CDC7 kinase activities in conjunction with other origin-firing factors convert some MCM-DHs into pairs of active CDC45-MCM-GINS helicases that nucleate bidirectional replisome establishment. (b) The prediction approach of ORI prediction using epigenomic marks, DNA motifs, and chromatin loops based on random forest classifier.

DNA replication in eukaryotic cells requires the accurate synthesis of large amounts of DNA, which is a critical factor that guarantees the fidelity of genetic information before cell division [1]. Errors in DNA replication can be amplified and accumulate over time, leading to cancer [2] and aging [3]. In eukaryotes, DNA replication starts from thousands of specific sites called the origin of DNA replication sites (ORIs), which are activated in a specified chronological order during each cell cycle [4]. The DNA replication initiation system mainly encompasses a highly regulated sequential two-step process (Figure 1(a)): origin licensing and origin activation [5]. The licensing and activation of replication origins are regulated by both DNA sequence and chromatin features [6]. It has been reported that, at each cell division in humans, 30,000-50,000 DNA replication origins are activated [7]. However, it is still unclear how they are selected and recognized by replication factors.

Recent studies also have certified that genomic and epigenomic characteristics contribute to the regulation of DNA replication initiation [8]. Eaton et al. found that chromatin modification helps to maintain the function and relative strength of replication initiation in Drosophila melanogaster genome [9]. Picard et al. emphasized that the coupling of H4K20me1 and H3K27me3 is associated with the improvement of replication origin efficiency of mammalian cell lines [10]. Long et al. showed that the histone variant H2A.Z epigenetically regulates the licensing and activation of early replication origins and maintains replication timing [11]. In addition, researchers have found a correlation between replication origin efficiency and chromatin architecture [12], as well as a linkage between replicons and chromatin loops [13]. More studies have revealed that the spatiotemporal replication initiation is regulated at the chromatin domain level [14]. These findings provided strong support and a basis for further study on the regulation mechanism, role, and function of epigenomic marks and chromatin structure on replication initiation.

Nowadays, a series of ORI identification algorithms based on machine learning or statistical analysis have emerged, such as iORI-PseKNC [15], iROS-gPseKNC [16], iRO-3wPseKNC [17], iOri-Human [18], Stack-ORI [19], yORIpred [20], iORI-ENST [21], ORI-Deep [22], Ori-Finder system [23], and iORI-Euk [24]. Unfortunately, these DNA-sequence-information-based approaches rarely elucidate the extent to which epigenomic marks, transcription factor (TF) motifs, and chromatin spatial structure regulate DNA replication initiation. Thus, we tested whether publicly available epigenome data, DNA motifs, and chromatin loop data can be used to mark human ORIs.

In this article, we demonstrated for the first time that ORIs can be computationally recognized using epigenomic marks, DNA motifs, and chromatin interactions (Figure 1(b)). Our model achieved excellent accuracy (AUC = 0.9033) by using available chromatin immunoprecipitation sequencing (ChIP-seq), deoxyribonuclease I- (DNase I-) hypersensitive site sequencing (DNase-seq), and reduced-representation bisulfite sequencing (RRBS-seq) data from ENCODE [25]. The top 20 features reflect the importance of chromatin accessibility, activity, and long-range contacts in determining ORIs. We also successfully predicted ORIs only using DNA motif occurrences (AUC = 0.9046) and identified the FOXL1 motif as a strong predictor. Moreover, a surprising high accuracy (AUC = 0.8488) was obtained only from the six-dimension features extracted from chromatin loop data. Furthermore, the combination of epigenomic marks, sequence-based TF motifs, and chromatin interactions exhibited a superior performance (AUC = 0.9627) for identifying ORIs compared with each single feature set. The results of feature analysis further revealed that the epigenome-based features, sequence-based features, and chromatin spatial structure-based features are informative and complementary in determining ORIs. We also found that the proposed iORI-Epi method in K562 cell line can be successfully applied to MCF7 and HCT116 cell lines, indicating that the method has good transferability in the recognition of ORIs.

## 2. Results

### 2.1. ORIs Can Be Predicted from Functional Genomic Features

**Figure 2:**
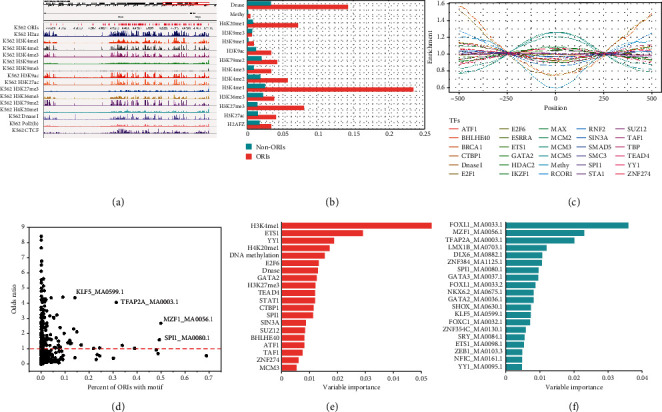
Epigenomic, chromatin, and DNA motif profiles of replication origin sites (ORIs). (a) A genome browser view of ORIs with histone marks, chromatin openness (DNase-seq), and DNA-binding proteins (e.g., CTCF). (b) Colocalization frequencies of histone modification, chromatin openness (DNase-seq), and DNA methylation at ORIs and non-ORIs. (c) Enrichment distribution of DNA-binding proteins at the ORI regions and ORI flanking regions. (d) Enrichment of DNA motifs at ORIs, as measured by the odds ratio and the percentage of ORI loci with a motif. (e) The top 20 variable importance of epigenomic marks. (f) The top 20 variable importance of DNA motifs.

The precise regulation of DNA replication initiation is a complex process involving many TFs and histone modifications (HMs). In order to qualitatively display the distribution of epigenomic mark signal in the replication initiation region, we applied the Integrative Genomics Viewer (IGV) [26] for visualization (Figure 2(a)). We found that most HM signals are strong in ORI-dense regions and weak in ORI-sparse regions, indicating that some HMs are colocalized and associated with active ORIs. In addition, compared with non-ORIs, the colocalization frequency of epigenomic marks was significantly enriched at ORIs (*p* < 0.01, *t*-test), except for H3K9me3 (*p* = 0.07, *t*-test), suggesting the high correlation between ORIs and epigenomic marks. The most abundant marks contained H3H4me1 and DNase I, which also reflects the high coupling between the initiation of replication, active epigenomic marks, and chromatin accessibility (Figure 2(b)). We also found that some TFs display significant enrichment between the ORI regions and ORI flanking regions (Figure 2(c)). Specifically, replication-related proteins (such as minichromosome maintenance (MCM) proteins) produce higher enrichment scores in the ORI region. This result implies that ChIP-seq data including epigenomic signals and DNase-seq data containing open chromatin marks in public databases are helpful to the selection of ORI regions.

**Figure 3:**
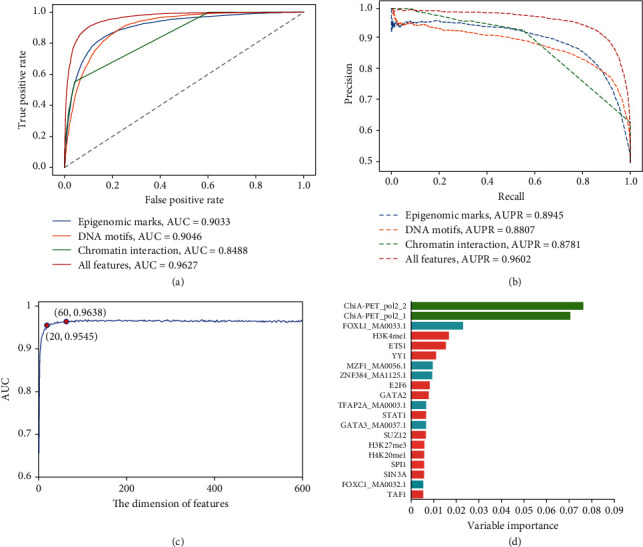
Prediction of ORIs using epigenomic data, DNA motifs, and ChIA-PET data by random forests. (a) Receiver operating characteristic (ROC) curve and (b) precision-recall (PR) curves for four different feature sets are plotted, in which area under the ROC curves (AUCs) and areas under the PR curves (AUPRCs) also are marked. (c) A plot showing the feature selection procedure for identifying ORIs based on 626-dimension features. When the top 60 features optimized by VI scores were used to perform prediction, the AUC nearly reaches IFS peak of 0.9638. At the same time, only the top 20 features can also produce a satisfactory model with an AUC value of 0.9545. (d) The top 20 variable importance values for all features included epigenomic marks, DNA motifs, and chromatin interaction.

In view of the close relationship between ORIs and epigenetic chromatin marks, we sought to build a classifier to distinguish ORIs from non-ORIs based on these marks. We collected ChIP-seq data of TFs and HMs commonly available to K562 cell line, DNase-seq, and RRBS-seq data from ENCODE to annotate ORIs and build feature vectors. Based on the random forest (RF) classifier, we obtained an excellent ORI prediction model with an AUC of 0.9033 (Figure 3(a)) and AUPRC of 0.8945 (Figure 3(b)).

In addition, the variable importance (VI) reflecting the contribution of marks as predictors was also calculated. Among the 20 most important epigenetic chromatin marks (Figure 2(e) and Table. S1), the histone marker H3K4me1 ranked first (VI = 0.0536), highlighting the significant role of active chromatin in replication initiation, as previously revealed by enrichment analysis. Moreover, it has been reported that the synergistic effect of H3k4me1 and H3K27me3 (VI = 0.0121, ranked 9th) makes the chromatin environment suitable for DNA replication initiation in the enhancer regions [27]. ETS1 protein (VI = 0.0291, ranked 2nd) also has great predictive importance for ORIs since the ETS family plays an essential role in the licensing of human MCM4 origin of replication [28]. In addition, they can activate transcription via binding to a core sequence located in the promoter elements [29], which indicate transcription and replication may share transcription factors in the two processes of occurrence. YY1 (VI = 0.0188, ranked 3rd) can bind replication-dependent histone genes to affect proliferation and chromatin remodeling to accelerate replication [30]. It also plays a major role in the coordinated upregulation of histone genes at the G1/S boundary of the cell cycle [31]. The fourth good marker H4K20me1 (VI = 0.0172) may affect the status of H4 acetylation, which can modulate origin of replication licensing [32]. DNA methylation (VI = 0.0154) and DNase I (VI = 0.0130) also performed good predictors, highlighting the roles of active chromatin and chromatin accessibility in predicting ORIs. The E2F transcription factors (VI = 0.0133, ranked 6th) are essential regulators of cell growth in multicellular organisms, controlling the expression of a number of genes whose products are involved in DNA replication and cell proliferation [33]. SUZ12 (VI = 0.0084, ranked 15th) is the subunit of PcG proteins, which has a high correlation with ORIs. Moreover, PcG and open chromatin marks have a synergistic effect in the selection of ORIs [27]. The epigenomic marks mentioned above are all related to DNA replication with higher VI scores, indicating that the features selected by RF are more explanatory and meaningful for ORI identification.

### 2.2. ORIs Can Be Predicted from DNA Motifs

We have predicted ORIs through epigenomic marks containing some TFs, but the ChIP-seq data of TFs available in public databases is limited, which means we cannot use more TF features as input of the model. Hence, we sought DNA motifs that may be enriched in ORIs as a way to obtain a more comprehensive list of candidate DNA-binding proteins. Among the 537 available motifs in the JASPAR 2018 database, 193 were significantly enriched (odd ratio > 1), indicating that ORIs are associated with a large number of protein binding sites (Figure 2(d)). Furthermore, 49 TFs of the C2H2 family accounted for the largest proportion of 193 TFs. C2H2 family has been shown to be more prone to binding to GC-rich motifs [34]. This finding is consistent with the conclusion that ORI of *H. sapiens* is located in GC-rich regions [24]. Among the most enriched and common motifs, SPI1 and MAF1 have significantly higher ORI percentages with prominent OR values (Figure 2(d)). The two motifs are both G-rich sequences. Investigations have discovered that SPI1 enhances the speed of DNA replication by acting particularly on elongation [35], and that MZF1 can activate the expression of MCM4 to promote the initiation of DNA replication [36]. That indicates they serve important roles in process of DNA replication.

Based on the above-mentioned DNA motif enrichment analysis, we explored the possibility of using the occurrences of DNA motifs to predict ORI. We built an RF classifier using 537 available motifs from the JASPAR 2018 database and obtained satisfactory prediction performance with an AUC of 0.9046 (Figure 3(a)) and AUPRC of 0.8807 (Figure 3(b)). Similarly, we also picked out the optimal 20 variables of DNA motifs according to their VI scores for further analysis (Figure 2(f) and Table. S1). We found that the fork-head box (FOX) family motifs (FOXL1_MA0033.1, ranked 1st; FOXL1_MA0033.2, ranked 9th; and FOXC1_MA0032.1, ranked 14th) showed optimistic predictors for ORI classification. Fox family TFs were demonstrated to play critical roles in regulating DNA replication and cell cycle, in which they can directly participate in DNA replication and determine the global replication timing program in a transcription-independent mechanism [37]. GATA family motifs (GATA3_MA0037.1, ranked 8th; GATA2_MA0036.1, ranked 11th) were also observed to have higher levels in the S phase of DNA replication [38], which explained their higher contribution to the ORI prediction model. This indicates that RF can select effective DNA motifs for ORI recognition.

### 2.3. ORIs Can Be Predicted from Chromatin Interaction

**Figure 4:**
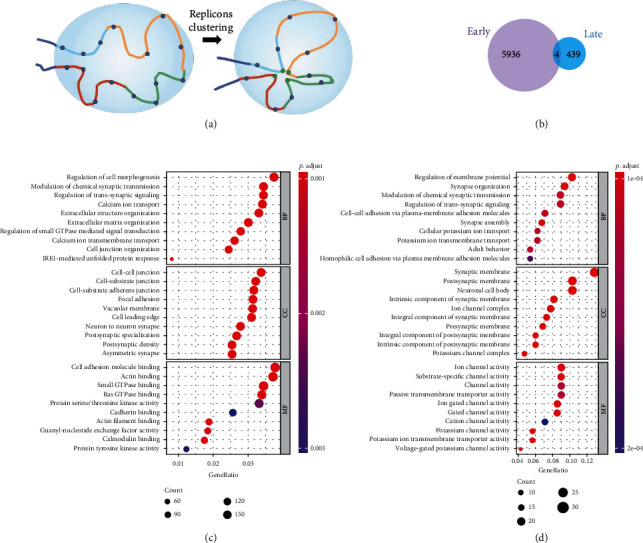
Analysis of properties and biological significance for early and late RT-state ORIs. (a) The schematic shows a chromatin domain containing four consecutive replication units (shown in different colors). Each replication unit contains three to four potential flexible replication origins (blue circles) on average. These replication units interact to form a replicon cluster in which the origins that will be activated (one per replication unit; green circles) gather together within the cluster. In a cluster, DNA replication origins that interact (green circles) fire synchronously and the cluster is identified as a replication focus in which ongoing DNA replication can be detected. (b) The number of specific genes to ORIs in early and late RT-states. (c, d) Bubble chart showing GO terms of the early ORIs and late ORIs in the category of biological process (BP), cellular component (CC), and molecular function (MF).

An important feature of ORIs is that their activation is usually synchronous in that several consecutive replication units form a replication cluster [39]. As shown in Figure 4(a), each replication unit (replisome) contains an average of three to four potential flexible replication origins (blue circles) [40]. These replication units (chromatin loop) interact to form a replication domain (RD), in which the selected ORIs will be synchronously activated (green circles) within the cluster by gathering at specific times during the S phase. The CoREP model pointed out that replication activation events may take place preferentially at CTCF-mediated loop anchors within each RD and then propagate to the periphery of the domain according to the observed spatiotemporal pattern of replication foci (RFi) propagation during early S phase [41]. Recently, the CRISI model was proposed to reveal a new ORI selection mechanism, in which multiple high-efficiency ORIs locate at the periphery of the topologically associating domains (TADs) at the beginning of S phase and are preferentially fired under the influence of the replication machinery protein PCNA [42]. Thus, the three-dimensional (3D) genome structure plays a regulatory role in ORI selection.

To investigate if chromosome conformation data could contribute to ORI prediction, ChIA-PET data [43] and Hi-C data [44] of K562 were collected. Subsequently, chromatin interaction abundance [45] and the overlapping ratio between anchors and ORIs were calculated to establish a model on the basis of RF classifier. Finally, a model with an AUC of 0.8488 (Figure 3(a)) and AUPR of 0.8781 (Figure 3(b)) was generated by six-dimension features.

In which, the features of RNA polymerase II- (Pol2A-) mediated loops were ranked first and second, respectively (Figure 3(d), green bar) in the 626-dimension fusion feature set described as below, indicating that chromatin interaction information is meaningful for the detection of ORIs. This result of the model confirmed that the information from chromatin interaction could provide effective help for the recognition of ORIs.

### 2.4. Feature Selection Strategy Is Significant for ORI Prediction

To further improve the predictive performance of the model, the extracted features from epigenomic marks, DNA motifs, and chromatin loops were combined to form a 626-dimension feature set. As shown in Figures 3(a) and 3(b), the model trained on the fusion feature set achieved better performance (AUC = 0.9627 and AUPR = 0.9602) when compared with the models trained on the single feature set, suggesting that the feature fusion strategy is effective in the detection of ORIs and could produce significant performance improvement.

However, heterogeneous features may lead to dimension disaster, bring noise, and reduce the robustness of the model, which may undermine model performance. To overcome these disadvantages, based on generated VI scores, the recursive feature elimination (RFE) technique [46] was applied to optimize the features. Our experiments showed the performance of the model still increased slightly (AUC from 0.9627 to 0.9638) when the feature dimension reduced from 626 to 60 (Figure 3(c)), which demonstrated that there was a lot of information redundancy and noise in the initial fusion features. In addition, the dimensions of the selected features are significantly lower than that of the original fusion feature set, which shortens the running time of the prediction model and saves computing resources (Table. S2). As the feature dimension is further reduced from 60 to 1, the prediction performance of the model showed a downward trend. The reason for the phenomenon is that fewer features cannot afford enough information of replication initiation. Thus, these results provide useful insights when considering building prediction models.

Then, the importance and contribution of features were further analyzed to find out which feature was more valuable to the model performance after feature selection. In the 60-dimension optimal feature set, the epigenetic mark-based features, sequence-based features, and three-dimension genome-based features account for 48.3%, 48.3%, and 3.3%, respectively. Although these screened epigenomic-based and sequence-based features are much more than the three-dimension genome-based features, they only account for 34.9% (29/83) of the total epigenomic marks and 5.4% (29/537) of the total sequence features. Accordingly, two features based on the chromatin interaction were with highest VI scores (Figure 3(d)), which means that the three-dimension genome features are new information independent of epigenomic and sequence, and are indispensable information in ORI recognition. If we further reduce the dimension of the optimal feature set to 20 features, the model can still produce an AUC of 0.9515. Among the top 20-rank features, 60.0% (12/20) were epigenomic mark-based features, 30.0% (6/20) were sequence-based features, and 10.0% (2/20) were chromatin interaction-based features (Figure 3(d)).

From the above analysis, it can be concluded that the feature encoding schemes used in the study are effective to improve the prediction ability of the model. In addition, the epigenomic information plays a more important role in the initiation of genome replication. We speculate that sequence-based features can be used as complementary information for epigenomic marks and chromatin interaction to identify ORIs.

### 2.5. The Different Strategies of Negative Class Prove Model Performance Is Not Inflated

To assess whether the high prediction accuracy of the model was overestimated due to the way we selected non-ORIs (the negative class), we designed the three different strategies on the basis of selected top 60 features.

**Figure 5:**
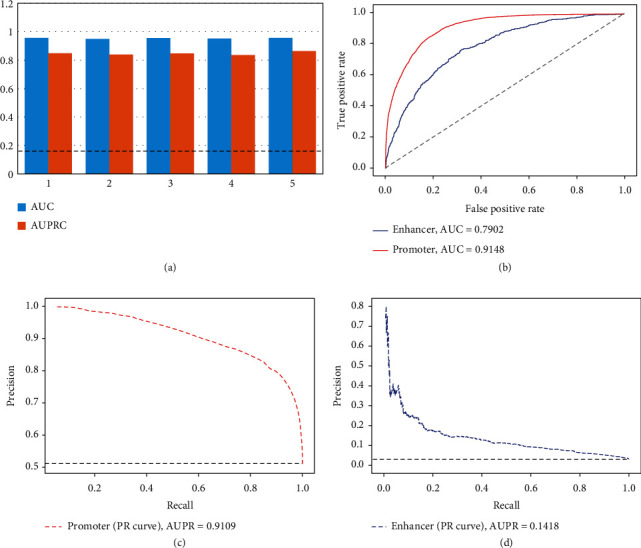
Different strategies of model validation by controlling negative class. (a) AUCs and AUPRCs for five imbalanced datasets of K562. The value of AUPR baseline is 0.1667. (b) ROC curves for ORI prediction in promoter and enhancer datasets. AUROCs are plotted. (c) PR curve for ORI prediction by using promoter data. The value of AUPR baseline is 0.5012. (d) PR curve for ORI prediction by using enhancer data. The value of AUPR baseline is 0.030.

First, we considered that the number of non-ORI regions far exceeds the number of ORI regions in the whole genome. Constructing a dataset with a lower positive to negative ratio could better reflect the reality. Therefore, positive samples were divided into five equal subpositive sets, and then, we combined them with whole negative samples to form five new datasets with a 1 : 5 ratio of positive and negative. On such datasets, our model could still produce very good AUCs (the average is 0.9536). The AUPRs (the average is 0.8473) were also much larger than the AUPR baseline of 0.1667 (Figure 5(a)).

Second, we focused on ORIs with gene promoter activity and built an RF classifier to discriminate ORIs associated with the promoters (35,977 sites) from promoters without ORIs (35,801 sites). The model could still achieve the satisfactory results (AUC = 0.9148 in Figure 5(b) and AUPR = 0.9109 in Figure 5(c)).

Third, we constructed a classifier to distinguish ORIs associated with enhancers (1,950 samples) from enhancers without ORIs (63,415 sites). Due to the high-class imbalance of data, we observed lower AUC (0.7902; Figure 5(b)) and AUPR (0.1418; Figure 5(d)), suggesting that false positives can be detected by our method. The three experiments suggested that the excellent accuracy of our model was not exaggerated by the selection of non-ORI strategy in the genome.

### 2.6. Independent Datasets Validate High Predictive Ability of Model

To further evaluate the predictive ability of the proposed model, we designed two independent datasets, which are separated from the benchmark dataset and downloaded from the Replication Domain database [47].

**Figure 6:**
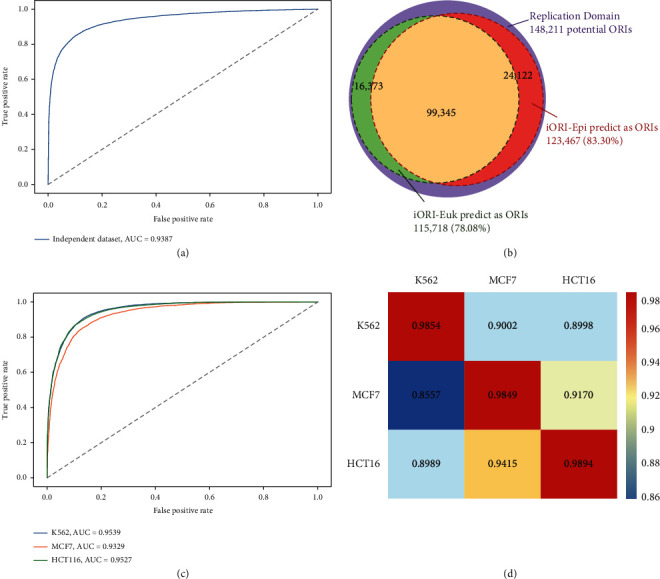
The analysis of the robustness and reliability of the model by independent dataset validation and cross-cell line validation. (a) ROC curve of independent datasets for K562 with the AUC of 0.9387. (b) 83.30% of 148,211 potential ORIs downloaded from Replication Domain database were predicted to be true ORIs based iORI-Epi. And 78.08% potential ORIs were predicted to be true ORIs by iORI-Euk. (c) The AUC values of K562, MCF-7, and HCT116 based on available 38-dimension features are 0.9539, 0.9329, and 0.9527, respectively. (d) The heat map shows the prediction performance in cross-cell line validation. Once a classification model of the cell line was established on its own dataset in columns, it was validated on its own data as well as another cell line data in rows.

The model to be verified was established based on the 60-dimension optimized features. As shown in Figure 6(a), the AUC of the model on the independent dataset is 0.9387, indicating that our model can effectively identify the ORIs. For the potential ORIs in Replication Domain database, we observed that our model could correctly identify 123,467 of 148,211 ORIs (83.30%) (Figure 6(b)). The accurate predictions of the above two experiments demonstrate that the model trained by 60-dimension features is a reliable strategy for recognizing ORIs.

Meanwhile, compared with our previous work that only used sequence information to predicted ORIs [24], iORI-Euk can recognize 115,718 of 148,211 ORIs (78.08%). Therefore, using only fewer dimensions of epigenomic marks, DNA motifs, and chromatin interaction may achieve better prediction outcomes.

### 2.7. The Proposed iORI-Epi Method Can Be Transferred to New Cell Lines

We further conducted cross-cell line validation using the knowledge of transfer information [48] to examine whether the model trained with K562 data could recognize the ORIs in other cell lines (here, we considered MCF7 and HCT116 cell lines, which were downloaded from GSE28911 [49]). The epigenomic information provided in the ENCODE database is different for different cell lines. Therefore, we need to select the intersection to obtain the epigenomic marks shared by the three cell lines. In the top 60 features, only 8 epigenomic marks were available for K562, MCF7, and HCT116 cell lines including 3 HMs, 3 TFs, DNA methylation, and DNase I (Table. S4). To generate the chromatin interaction features, we downloaded loop data for MCF7 and HCT116 from GEO database (GSE39495) and ENCODE (ENCFF246ZKR), respectively.

As a result, a total of 39-dimension fusion features were extracted from ORI benchmark datasets for the three cell lines. Using these features, we rebuilt the models on K562, MCF7, and HCT116 data and then yielded AUCs of 0.9539, 0.9329, and 0.9527, respectively (Figure 6(c)). Subsequently, we used any model to predict the datasets from other cell lines, and the obtained AUC heat map is shown in Figure 6(d) to describe the prediction performance of cross-cell line validation. From the heat map, it can be observed that all AUCs are greater than 0.85; that is, the ORI prediction across-cell line is successful. That also indicates our proposed method can be transferred to a dataset of other cell lines.

And then, based on the above-mentioned three models, the potential ORIs of the other two cell lines were used as independent sets, respectively. The cutoff value of prediction probability is set as 0.5, which means that prediction accuracy greater than 0.5 is regarded as true ORIs. We found that the probabilities of correctly predicting ORIs were greater than 70% (Figure S2a), suggesting that a model from one cell line can be used to identify the ORIs of another cell line to some extent.

Next, we wondered whether only using epigenetic marks and chromatin interaction features could also produce better performance. To this end, we eliminated the 39 sequence features, retained 8 epigenetic marks and 2 chromatin interaction features, and repeated the above prediction process. Finally, AUCs (0.8023, 0.8032, and 0.7730) and AUPRs (0.8454, 0.8266, and 0.8197) were generated on K562, MCF7, and HCT116 benchmark datasets, respectively (Figure S2b and S2c). And all calculated AUC values were greater than 0.54 in the heat map of cross-cell line (Figure S2d). This suggests that feature set integrating epigenetic marks, chromatin interactions, and sequence features contains more valuable information and is more conducive to generating robust models than single types of features. It also reflects that epigenomic marks, chromatin interaction information, and sequence features all play important regulatory roles in replication initiation.

### 2.8. GO Term Enrichment Analysis in Early ORIs and Late ORIs

The replication of eukaryotic chromosomes takes place in segments that generally replicate in a predictable temporal order, which is known as the replication timing (RT) program [50]. The RT program is related to many key biological processes, including cell fate commitment, the 3D structure of chromosomes, and transcription regulation. However, the biological significance of RT remains a puzzle [51].

To investigate whether there are specific pathways or gene sets that are enriched in ORIs of different RT states, we conducted GO term enrichment analysis on the genes overlapping with each of ORIs in early and late RT states [52]. According to statistics, the specific genes that overlap with early-state and late-state ORIs are 5,936 and 439, respectively (Figure 4(b)). This indicated that the gene dose of early replication initiation genes is higher than that of late replication initiation genes. Next, Figures 4(c) and 4(d) show the 10 most significantly enriched GO terms in each ontology group of early-state and late-state ORIs, respectively. We found that early ORIs are mainly involved in the process of modulating the frequency, rate, or extent of cell morphogenesis at the cellular level, such as regulation of cell morphogenesis, extracellular structure organization, extracellular matrix organization, cell junction organization, while late ORIs are enriched with GO terms related to stress response or defense response and are enriched in processes involving in neural cell regulation and tissue development.

### 2.9. Enrichment Analysis between Promoter-Related TFs and ORIs

Both DNA replication and gene transcription occur in the active chromatin compartments, and double-stranded DNA needs to be unraveled, raising a pivotal question, that is, whether these processes will share transcription factors to save resources. Previous studies have suggested that transcription may affect the initiation of DNA replication, but the underlying mechanism of this interaction in mammalian cells remains elusive [53, 54]. In general, as the binding sites of RNA polymerase (RNApol), promoters are usually located near the transcription start site (TSS) to turn genes on or off [55]. The eukaryotic promoter region is rich in a variety of motifs, mainly including TATA/TBP, ETS family, E2A family, SP1-like, NRF, and CREB/ATF [56].

**Figure 7:**
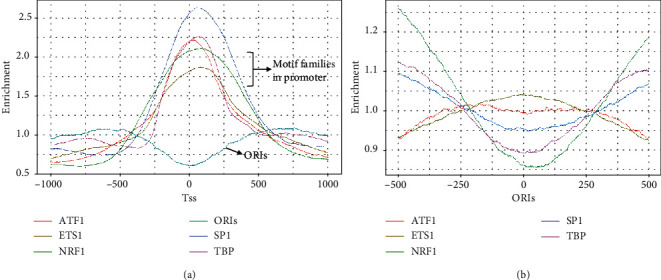
Enrichment distribution of cooccurring TFs in promoter regions on TSS regions (a) and ORI regions (b).

To investigate whether TFs bound on promoter are related to DNA replication initiation, we plotted the enrichment distributions of several major TFs on TSS and ORI (Figure 7(a)). We found that promoter-related TFs are intensely enriched in TSS locations compared with TSS flanking regions. In addition, ORIs displayed a significantly lower enrichment score at the TSS locations. Moreover, ATF1 and ETS1 showed significant enrichment in the ORI regions (Figure 7(b)). According to previous studies, the bZIP transcription factor (ATF1) is an activator of the anaphase promoting complex and facilitates degradation of the mitotic cyclin Cdc13 and the securin Cut2 [57]. ETS transcription factors are novel regulators of MCM4 origin, whose binding sites are localized between two divergently transcribing MCM4 and PRKDC genes [28]. Therefore, these two TFs play an important role in the process of transcription and replication.

Our findings imply that there is a chronological relationship between replication and transcription, and fewer TFs can participate in more biological processes to improve efficiency. Recently, Liu et al. proposed a “transcription bulldozing” model to describe the key role of transcription in maintaining genomic stability during DNA replication initiation in mammalian cells [58]. Therefore, mammalian cells employ an extremely sophisticated and multilayered coregulation mechanism to replicate and transcript in a highly coordinated manner.

## 3. Discussion

Various genetic and epigenomic signatures, including CpG islands, G-quadruplexes, nucleosome-depleted regions, and histone modifications, have been found to be associated with the initiation of DNA replication in eukaryotes [59]. Hence, it is obviously insufficient to decipher the mechanism of replication initiation selection only by DNA sequence information. For the first time, various functional genomics data, including ChIP-seq data of transcription factors and histone modifications, DNase-seq data of chromatin accessibility, RRBS-seq data of DNA methylation, and chromatin loops, were used to predict ORIs in this study. The method named iORI-Epi displayed excellent prediction accuracy in training dataset. Moreover, the prediction results on other cell lines and independent datasets also indicate that our model is highly scalable. We believe that iORI-Epi could serve as a useful tool for the discovery of novel ORIs and pave the way for a better understanding of DNA replication initiation.

To explore the conservative pattern of epigenome preference for DNA replication initiation, the ChIP-seq peaks of histone modification and transcription factors in K562 cell line from the ENCODE database were mapped to corresponding ORI regions. Previous research has shown that H3K9me3 has the highest level during and just after replication in HeLa S3 cells, in which H3K9me3 may be required for the regulation of replication at both heterochromatin and euchromatin regions [60]. However, we found that H3K9me3 is not significantly distributed in the ORI regions from IGV map (Figure 2(a)) and colocalization frequencies (Figure 2(b)). The genome-wide ORI distribution also showed that H3K9me3 had no significant signal both in the ORI-rich region of K562 and MCF-7 cell lines (Figure S3). Additionally, the IV value of H3K9me3 (VI = 5.61*e* − 05) also reflected that it has almost no contribution to the classification model. These results suggest that the initiation of DNA replication may be cell-specific, which also lays the foundation for the study of the cell-specific mechanism of ORI.

The enrichment distribution of DNA-binding proteins in the ORI regions and ORI flanking regions also showed an interesting distribution trend, in which DNA-binding proteins were symmetrically distributed almost centered on the midpoint of ORI (Figure 2(c)). All lines intersect at ±250 bp positions, forming a distinct ORI region and ORI flanking region. Therefore, we speculated that the length of ORI may be less than 500 bp. In fact, approximately 85% of ORIs were less than 500 bp in length for K562 cell line (Figure S1a), which exactly supports our conjecture. We also found that three cell lines (K562, MCF7, and HCT116) have similar distribution of length and distance between two adjacent ORIs, which all obey the gamma distribution [61] (Figure S4). This is a very interesting discovery, which provides a fundamental principle for the study of ORIs in human cell.

Moreover, we also propose several directions worth exploring in the future. Firstly, in this work, an epigenome-based model was constructed to predict ORIs, and satisfactory performance and computational efficiency were achieved. Since the initiation of eukaryotic genome replication is such a sophisticated process, it makes sense to apply the information from epigenomic signals and DNA-binding proteins to target specific ORIs. As a result, our computational methodology can be further extended to ORI identification in other eukaryotic species, tissues, and cell lines. Second, RNA polymerase II can redistribute MCM complexes to nontranscribed regions to minimize replication-transcription collisions and maintain genome stability in mammalian cells [58]. Therefore, it is worth probing into the relationship between transcription and replication selection. Third, here, we observed that chromatin loop anchors are important in predicting ORIs (Figure 3(d)). It is necessary to extract more features from 3D genome to identify ORIs and explain how the replication initiation event is spatially regulated in a replication domain.

## 4. Materials and Methods

### 4.1. Benchmark Dataset Construction

We collected K562 ORIs with genomic location in BED format from GSE28911 [49] as positive samples, the replication initiation profiles obtained through massively parallel sequencing of nascent DNA strands. Meanwhile, the length distribution of ORIs (Figure S1a) and the distance distribution between two adjacent ORIs (Figure S1b) indicated more than approximately 95% of ORIs between 100 bp and 800 bp in length, and more than 60% of the adjacent ORIs were less than 10,000 bp. Therefore, the sequence fragments with length in ranges of 800-1000 bp located between the adjacent ORIs with length more than 10,000 bp were selected as on-ORI samples.

Generally, independent datasets should be established for objectively evaluation proposed model. Therefore, we divided the benchmark dataset into training dataset and independent dataset in a ratio of 7 : 3 in both positive samples and negative samples (Figure S1c). The training dataset was used to build classification model, in which 8/10 is used to train the model and 2/10 is used to test and tune the model. Once the model is determined, the independent dataset was applied to further validate the model. In addition, the data of K562 ORIs is also collected from another database called Replication Domain [47] to generate the second independent dataset to test the performance of the model.

### 4.2. Encoding Schemes

We downloaded 83 epigenomic marks with bed format from the ENCODE for K562 cell line, which included 69 TF binding profiles, 12 HMs, chromatin accessibility, and DNA methylation (Table S2). The human genome assembly hg19 was as our reference. We calculated the overlap ratio of epigenomic marks with ORI and non-ORI regions as feature vectors.

We also downloaded 537 DNA motifs of transcription factor binding sites from the JASPAR 2018 database [62]. According to the corresponding position weight matrices, we can call these motifs over DNA sequences using a minimum matching score of 80%. Therefore, the number of motif occurrences within ORI and non-ORI regions was calculated.

As for the part of chromatin interaction features, the ChIA-PET data was download from NCBI/GEO, which included Pol2A-mediated loop data (GSM970213) and CTCF-mediated loop data (GSM970216). The Hi-C data was collected from Rao's study (GSE63525). For each loop data, the number of ChIA-PET/Hi-C interactions that have overlapping with the ORI regions at both ends was used as the feature of chromatin interaction abundance. We also calculated the overlap ratio between loop anchors and ORI regions.

### 4.3. Machine Learning

The random forest (RF) algorithm is a flexible and practical machine learning method based on bagging, which consists of a large number of individual decision trees that operate as an ensemble. Here, we used an R package ranger to execute RF classifier with default package parameters. Meanwhile, the feature importance score was calculated using the mean decrease in accuracy in the out-of-bag sample. Thus, the features were ranked from large to small according to the obtained feature importance score, and then, the best feature subset that can produce the best prediction performance is obtained by the recursive feature elimination (RFE) technique [46].

## Data Availability

We provide the benchmark datasets and source code used in this study, which are freely available in the GitHub repository (https://github.com/linDing-group/iORI-Epi).

## Supplementary Materials

Supplementary Materials Figure S1: the information of ORIs in human K562. (a) The length distribution of ORIs. (b) The distance distribution between two adjacent ORIs. (c) The benchmark dataset was separated training dataset to build model and independent dataset to validate model in a ratio of 7 : 3. Figure S2: (a) a model of one cell line obtained by 39-dimension features was used to identify the potential ORIs of the other two cell lines. (b, c) The analysis of the robustness and reliability of model based on 8 epigenomic marks and 2 chromatin interaction features. The AUC and AUPR values of K562, MCF7, and HCT116 based on 10-dimension features are recorded, respectively. (d) The heat map showing the prediction performance in cross-cell line validation based on 10-dimension epigenetic marks and chromatin interaction. Once a classification model of cell line was established on its own dataset in columns, it was validated on its own data as well as another cell line data in rows. Figure S3: a genome browser view of ORIs with H3K9me3 signal in whole genome for K562 and MCF-7 Figure S4. The length distribution of ORIs (left) and the distance distribution between two adjacent ORIs (right) in K562, MCF7, and HCT116 cell lines. Table S1: functional annotations on DNA replication-related HMs and TFs in the top 20 features. Table S2: the 83 epigenomic marks were downloaded from the ENCODE for K562 cell line. Table S3: time of growing tree model and computing permutation importance for different feature dimensions. Table S4: in the top 60 features, 8 epigenomic marks including 3 histone marks, 3 transcription factors, DNA methylation, and DNase I were downloaded from ENCODE for MCF7 and HCT116 cell lines.
