# Supplementary material for: Accurate Identification of DNA Replication Origin by Fusing Epigenomics and Chromatin Interaction Information
Source: Research (Wash D C). 2022 Oct 29;2022:9780293. doi: 10.34133/2022/9780293 (PMC9667886; doi:10.34133/2022/9780293)
Supplement: Supplementary Materials — Figure S1: the information of ORIs in human K562. (a) The length distribution of ORIs. (b) The distance distribution between two adjacent ORIs. (c) The benchmark dataset was separated training dataset to build model and independent dataset to validate model in a ratio of 7 : 3. Figure S2: (a) a model of one cell line obtained by 39-dimension features was used to identify the potential ORIs of the other two cell lines. (b, c) The analysis of the robustness and reliability of model based on 8 epigenomic marks and 2 chromatin interaction features. The AUC and AUPR values of K562, MCF7, and HCT116 based on 10-dimension features are recorded, respectively. (d) The heat map showing the prediction performance in cross-cell line validation based on 10-dimension epigenetic marks and chromatin interaction. Once a classification model of cell line was established on its own dataset in columns, it was validated on its own data as well as another cell line data in rows. Figure S3: a genome browser view of ORIs with H3K9me3 signal in whole genome for K562 and MCF-7 Figure S4. The length distribution of ORIs (left) and the distance distribution between two adjacent ORIs (right) in K562, MCF7, and HCT116 cell lines. Table S1: functional annotations on DNA replication-related HMs and TFs in the top 20 features. Table S2: the 83 epigenomic marks were downloaded from the ENCODE for K562 cell line. Table S3: time of growing tree model and computing permutation importance for different feature dimensions. Table S4: in the top 60 features, 8 epigenomic marks including 3 histone marks, 3 transcription factors, DNA methylation, and DNase I were downloaded from ENCODE for MCF7 and HCT116 cell lines. [file 9780293.f1.docx]

Supplementary Materials for

Accurate Identification of DNA Replication Origin by Fusing Epigenomics and Chromatin Interaction Information

Fu-Ying Dao^1,2,3^, Hao Lv^1,4^, Melissa J. Fullwood^2,3,5*^ and Hao Lin^^[[1]](#footnote-1)^*^

^1^ Center for Informational Biology, University of Electronic Science and Technology of China, Chengdu 610054, China;

^2^ School of Biological Sciences, Nanyang Technological University, Singapore 639798, Singapore;

^3^ Cancer Science Institute of Singapore, National University of Singapore, 14 Medical Dr, Singapore 117599, Singapore;

^4^ Department of Molecular Life Sciences, University of Zurich, Winterthurerstrasse 190, 8057 Zurich, Switzerland;

^5^ Institute of Molecular and Cell Biology, Agency for Science, Technology and Research (A*STAR), Singapore 138673, Singapore.


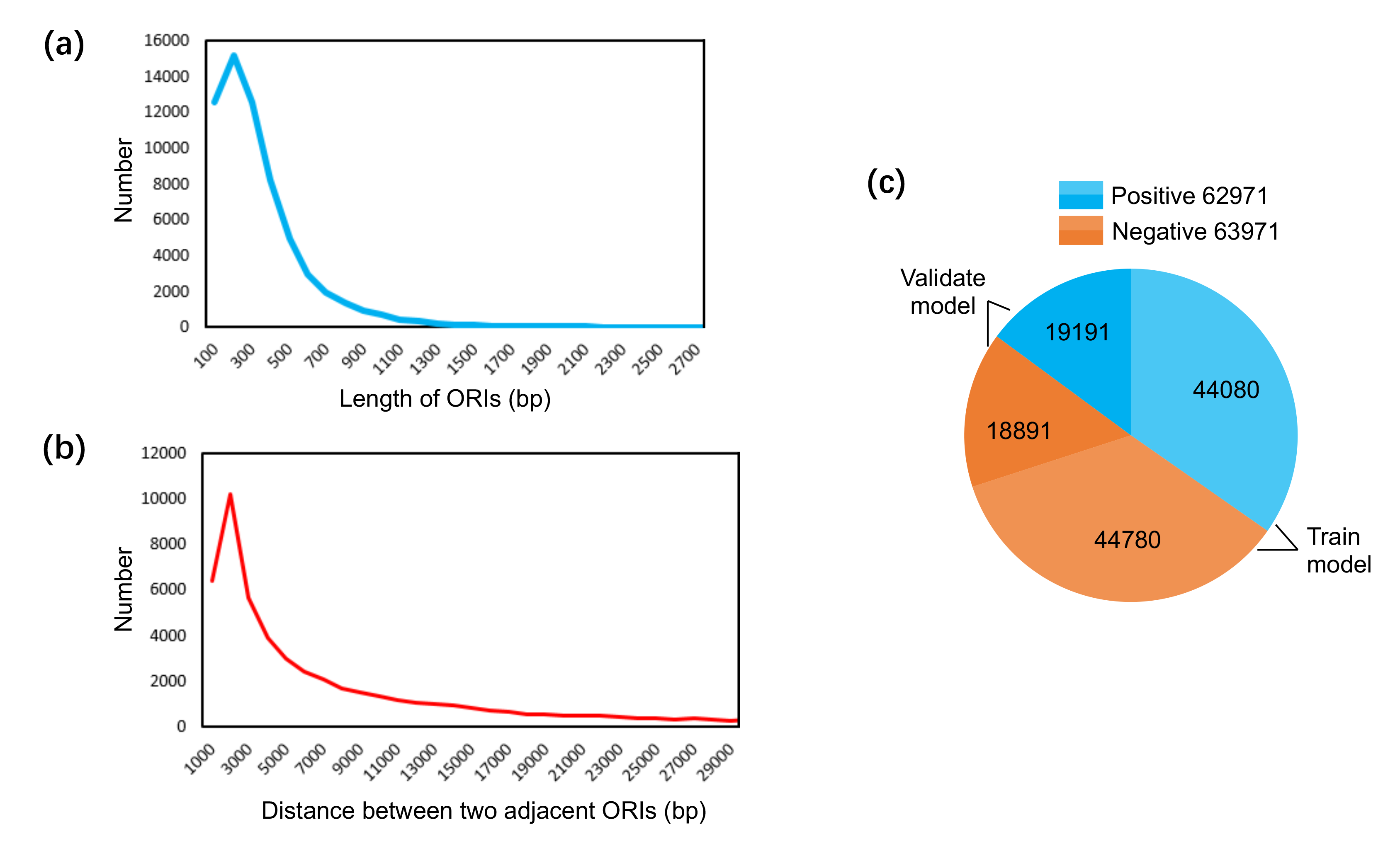


Fig. S1. The information of ORIs in human K562. (a) The length distribution of ORIs. (b) The distance distribution between two adjacent ORIs. (c) The benchmark dataset was separated training dataset to build model and independent dataset to validate model in a ratio of 7:3.


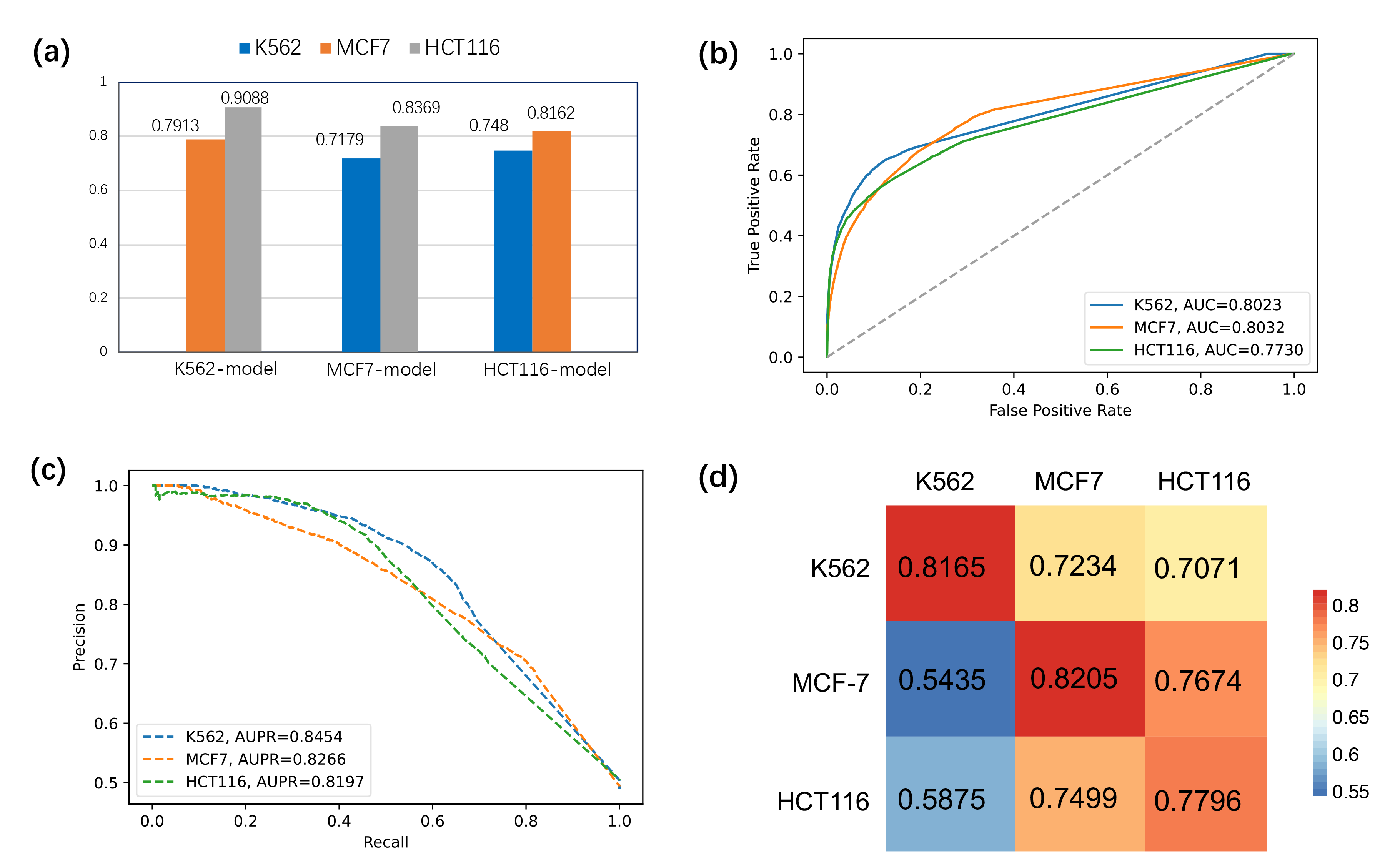


Fig. S2. (a) A model of one cell line obtained by 39-dimension features was used to identify the potential ORIs of the other two cell lines. (b, c) The analysis of the robustness and reliability of model based on 8 epigenomic marks and 2 chromatin interaction features. The AUC and AUPR values of K562, MCF7, and HCT116 based on 10- dimension features are recorded, respectively. (d) The heat map showing the prediction performance in cross-cell line validation based on 10-dimension epigenetic marks and chromatin interaction. Once a classification model of cell line was established on its own dataset in columns, it was validated on its own data as well as another cell line data in rows. Type or paste caption here. Create a page break and paste in the Figure above the caption.

**
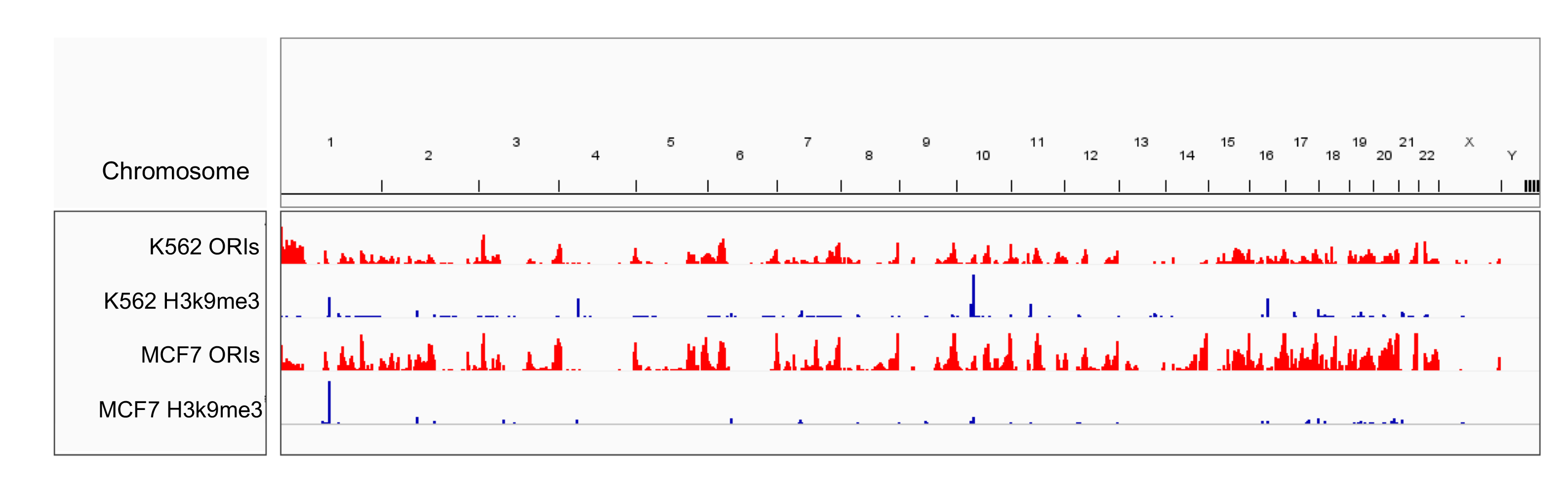
**

Fig. S3. A genome browser view of ORIs with H3K9me3 signal in whole genome for K562 and MCF-7


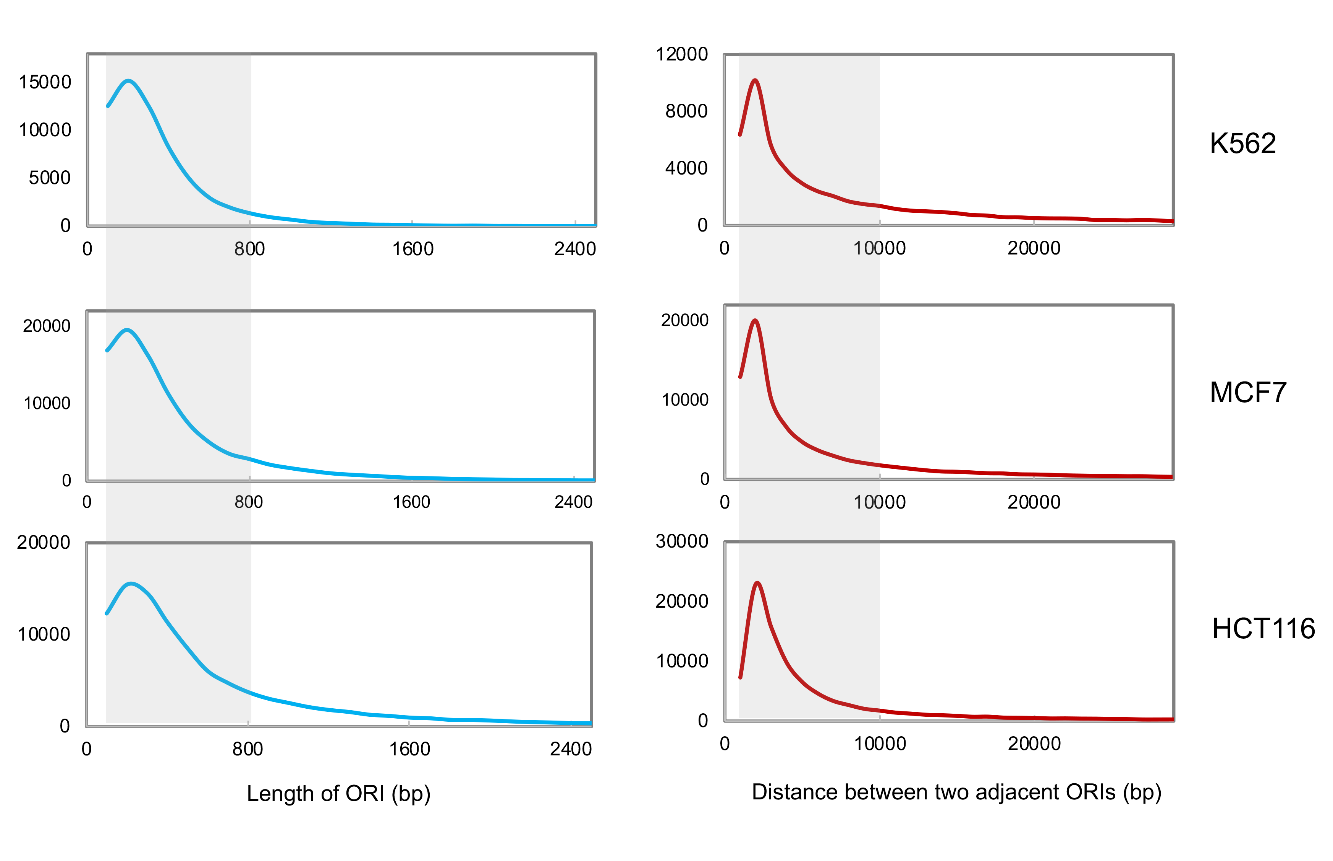


Fig. S4. The length distribution of ORIs (left) and the distance distribution between two adjacent ORIs (right) in K562, MCF7 and HCT116 cell lines.

Table S1. Functional annotations on DNA replication-related HMs and TFs in the top 20 features.

| HMs and TFs | Functional annotation | Refs. |
| --- | --- | --- |
| H3K4me1/ H3K27me3 | Cooperation of H3K4me1 and H3K27me3 to allow a chromatin environment suitable to initiate replication in some enhancer regions. | [1] |
| H4K20me1 | H4K20me1 may affect the status of H4 acetylation, which can modulate origin of replication licensing. | [2] |
| ETS1 | ETS family plays an essential role in the licensing of human MCM4 origin of replication. | [3, 4] |
| YY1 | YY1 plays a major role in the coordinate up-regulation of histone genes at the G1/S boundary of the cell cycle. | [5] |
| E2F | E2F are essential regulators of cell growth in multicellular organisms, controlling the expression of a number of genes whose products are involved in DNA replication and cell proliferation. | [6] |
| SUZ12 | SUZ12 is the subunite of PcG proteins, which has a high correlation with ORIs. Moreover, PcG and open chromatin marks have a synergistic effect in the selection of ORIs | [1] |
| FOXL1 | Fox family TFs were demonstrated to play critical roles in regulating DNA replication and cell cycle, in which they can directly participate in DNA replication and determine the global replication timing program in a transcription-independent mechanism. | [7] |
| GATA3 | GATA family TFs were also observed to have higher levels in the S phase of DNA replication. | [8] |

Table S2. The 83 epigenomic marks were download from the ENCODE for K562 cell line.

| Target | Accession | Assay |
| --- | --- | --- |
| ARNT | ENCFF333QJD | TF ChIP-seq |
| ATF1 | ENCFF089SHU | TF ChIP-seq |
| ATF2 | ENCFF826QEY | TF ChIP-seq |
| ATF7 | ENCFF611BUY | TF ChIP-seq |
| BACH1 | ENCFF276CQA | TF ChIP-seq |
| BHLHE40 | ENCFF159GKF | TF ChIP-seq |
| BRCA1 | ENCFF248TTW | TF ChIP-seq |
| CDC5L | ENCFF108JTM | TF ChIP-seq |
| CTBP1 | ENCFF066ESG | TF ChIP-seq |
| CTCF | ENCFF545EHA | TF ChIP-seq |
| CUX1 | ENCFF725FYI | TF ChIP-seq |
| E2F1 | ENCFF260KBY | TF ChIP-seq |
| E2F6 | ENCFF072IAO | TF ChIP-seq |
| E4F1 | ENCFF952KDF | TF ChIP-seq |
| EGR1 | ENCFF637AOG | TF ChIP-seq |
| ELF4 | ENCFF625PVR | TF ChIP-seq |
| ELK1 | ENCFF390VNW | TF ChIP-seq |
| EP300 | ENCFF721DKV | TF ChIP-seq |
| ESRRA | ENCFF106AUG | TF ChIP-seq |
| ETS1 | ENCFF128BVU | TF ChIP-seq |
| FOXA1 | ENCFF837QWL | TF ChIP-seq |
| FOXM1 | ENCFF804EPQ | TF ChIP-seq |
| GATA1 | ENCFF234GQW | TF ChIP-seq |
| GATA2 | ENCFF717ZNF | TF ChIP-seq |
| GTF2F1 | ENCFF846SYJ | TF ChIP-seq |
| HDAC1 | ENCFF080VTW | TF ChIP-seq |
| HDAC2 | ENCFF368EUJ | TF ChIP-seq |
| IKZF1 | ENCFF165OLG | TF ChIP-seq |
| JUN | ENCFF422SBX | TF ChIP-seq |
| KDM1A | ENCFF499UMV | TF ChIP-seq |
| KLF16 | ENCFF373FDW | TF ChIP-seq |
| MAFK | ENCFF660EKS | TF ChIP-seq |
| MAX | ENCFF417GYT | TF ChIP-seq |
| MCM2 | ENCFF834QOU | TF ChIP-seq |
| MCM3 | ENCFF530ETK | TF ChIP-seq |
| MCM5 | ENCFF501SOY | TF ChIP-seq |
| MCM7 | ENCFF191CDX | TF ChIP-seq |
| MNT | ENCFF595GHN | TF ChIP-seq |
| MXI1 | ENCFF400DMX | TF ChIP-seq |
| MYC | ENCFF165VLE | TF ChIP-seq |
| NFIC | ENCFF754CMK | TF ChIP-seq |
| NRF1 | ENCFF477OCU | TF ChIP-seq |
| POLR2A | ENCFF763UDO | TF ChIP-seq |
| POLR2AphosphoS2 | ENCFF039RTS | TF ChIP-seq |
| POLR2AphosphoS5 | ENCFF065RJC | TF ChIP-seq |
| RAD21 | ENCFF002CXU | TF ChIP-seq |
| RCOR1 | ENCFF620JKI | TF ChIP-seq |
| RFX5 | ENCFF066GNV | TF ChIP-seq |
| RNF2 | ENCFF134OWN | TF ChIP-seq |
| SIN3A | ENCFF877VHC | TF ChIP-seq |
| SMAD5 | ENCFF235FTG | TF ChIP-seq |
| SMC3 | ENCFF892UIH | TF ChIP-seq |
| SP1 | ENCFF103ESU | TF ChIP-seq |
| SPI1 | ENCFF507MNQ | TF ChIP-seq |
| STAT1 | ENCFF473ULH | TF ChIP-seq |
| STAT2 | ENCFF697GQV | TF ChIP-seq |
| STAT5A | ENCFF556KAN | TF ChIP-seq |
| SUZ12 | ENCFF731WWO | TF ChIP-seq |
| TAF1 | ENCFF697OMD | TF ChIP-seq |
| TAL1 | ENCFF435YKY | TF ChIP-seq |
| TARDBP | ENCFF612BYB | TF ChIP-seq |
| TBP | ENCFF891AHH | TF ChIP-seq |
| TCF12 | ENCFF334SEU | TF ChIP-seq |
| TEAD4 | ENCFF318EUW | TF ChIP-seq |
| USF1 | ENCFF738UPC | TF ChIP-seq |
| YY1 | ENCFF228AYO | TF ChIP-seq |
| ZBTB33 | ENCFF108BVL | TF ChIP-seq |
| ZNF143 | ENCFF971SGR | TF ChIP-seq |
| ZNF274 | ENCFF565OZP | TF ChIP-seq |
| DNA Methylation | ENCFF001TOL | RRBS-seq |
| DNase I | ENCFF162UKK | DNase-seq |
| H3K9me1 | [ENCFF374ISV](https://www.encodeproject.org/files/ENCFF374ISV/) | Histone ChIP-seq |
| H3K9me3 | [ENCFF388GQN](https://www.encodeproject.org/files/ENCFF388GQN/) | Histone ChIP-seq |
| H3K4me1 | [ENCFF564QIG](https://www.encodeproject.org/files/ENCFF564QIG/) | Histone ChIP-seq |
| H3K4me2 | [ENCFF627TIZ](https://www.encodeproject.org/files/ENCFF627TIZ/) | Histone ChIP-seq |
| H3K4me3 | [ENCFF752ALB](https://www.encodeproject.org/files/ENCFF752ALB/) | Histone ChIP-seq |
| [H2AFZ](https://www.encodeproject.org/targets/H2AFZ-human/) | [ENCFF681OSA](https://www.encodeproject.org/files/ENCFF681OSA/) | Histone ChIP-seq |
| [H4K20me1](https://www.encodeproject.org/targets/H4K20me1-human/) | [ENCFF189YWV](https://www.encodeproject.org/files/ENCFF189YWV/) | Histone ChIP-seq |
| [H3K79me2](https://www.encodeproject.org/targets/H3K79me2-human/) | [ENCFF350GQM](https://www.encodeproject.org/files/ENCFF350GQM/) | Histone ChIP-seq |
| [H3K27ac](https://www.encodeproject.org/targets/H3K27ac-human/) | [ENCFF931VAQ](https://www.encodeproject.org/files/ENCFF931VAQ/) | Histone ChIP-seq |
| [H3K9ac](https://www.encodeproject.org/targets/H3K9ac-human/) | [ENCFF418BIN](https://www.encodeproject.org/files/ENCFF418BIN/) | Histone ChIP-seq |
| [H3K36me3](https://www.encodeproject.org/targets/H3K36me3-human/) | [ENCFF784HLI](https://www.encodeproject.org/files/ENCFF784HLI/) | Histone ChIP-seq |
| H3K27me3 | [ENCFF908KJV](https://www.encodeproject.org/files/ENCFF908KJV/) | Histone ChIP-seq |

Table S3. Time of growing tree model and computing permutation importance for different feature dimensions.

| Feature dimension | Growing tree | Computing permutation importance |
| --- | --- | --- |
| 626-D | 3 minutes 10 seconds | 57 minutes 45 seconds |
| 60-D | 13 seconds | 42 seconds |

Table S4. In the top-60 features, 8 epigenomic marks including 3 histone marks, 3 transcription factors, DNA methylation, and DNase I were downloaded from ENCODE for MCF7 and HCT116 cell lines.

| Feature | MCF7-Accession | HCT116-Accession | Assay | VI score |
| --- | --- | --- | --- | --- |
| H3K4me1 | ENCFF191EBN | ENCFF168GCU | Histone ChIP-seq | 0.0129 |
| SIN3A | ENCFF808QLK | ENCFF493SWH | TF ChIP-seq | 0.0064 |
| H3K27me3 | ENCFF305ASW | ENCFF029ZPV | Histone ChIP-seq | 0.0067 |
| DNA Methylation | ENCFF001TPA | ENCFF001TMN | RRBS-seq | 0.0053 |
| H4K20me1 | ENCFF959AXO | ENCFF232MXI | Histone ChIP-seq | 0.0067 |
| TEAD4 | ENCFF000QRR | ENCFF008LFE | TF ChIP-seq | 0.0046 |
| DNase I | ENCFF846GPW | ENCFF228YKV | DNase-seq | 0.0051 |
| MAX | ENCFF169IXS2 | ENCFF214KLO | TF ChIP-seq | 0.0027 |

References

[1] Cayrou C, Ballester B, Peiffer I, et al. The chromatin environment shapes DNA replication origin organization and defines origin classes. Genome Res, 2015, 25: 1873-1885

[2] Miotto B, Struhl K. Hbo1 histone acetylase activity is essential for DNA replication licensing and inhibited by geminin. Mol Cell, 2010, 37: 57-66

[3] Sidhu K, Kumar V. C-ets transcription factors play an essential role in the licensing of human mcm4 origin of replication. Biochim Biophys Acta, 2015, 1849: 1319-1328

[4] Seth A, Robinson L, Thompson DM, et al. Transactivation of gata-1 promoter with ets1, ets2 and ergb/hu-fli-1 proteins: Stabilization of the ets1 protein binding on gata-1 promoter sequences by monoclonal antibody. Oncogene, 1993, 8: 1783-1790

[5] Huang NE, Lin CH, Lin YS, et al. Modulation of yy1 activity by sap30. Biochem Biophys Res Commun, 2003, 306: 267-275

[6] Hateboer G, Wobst A, Petersen BO, et al. Cell cycle-regulated expression of mammalian cdc6 is dependent on e2f. Mol Cell Biol, 1998, 18: 6679-6697

[7] Jin Y, Liang Z, Lou H. The emerging roles of fox family transcription factors in chromosome replication, organization, and genome stability. Cells, 2020, 9:

[8] Merika M, Orkin SH. DNA-binding specificity of gata family transcription factors. Mol Cell Biol, 1993, 13: 3999-4010

1. * Corresponding author.

   Hao Lin: hlin@uestc.edu.cn, Tel: (86)13678168394;

   Melissa J. Fullwood: mfullwood@ntu.edu.sg, Tel: (65)65162178 [↑](#footnote-ref-1)
